# Supplementary material for: Factors associated with receipt of adequate antenatal care among women in Rwanda: A secondary analysis of the 2019–20 Rwanda Demographic and Health Survey
Source: PLoS One. 2023 Apr 20;18(4):e0284718. doi: 10.1371/journal.pone.0284718 (PMC10118085; doi:10.1371/journal.pone.0284718)
Supplement: Attachment — Submitted filename: Rebuttal letter.docx [file pone.0284718.s001.docx]

#### Rebuttal letter

Dear Editor,

Thank you for taking the time to review our manuscript and allow us the opportunity to respond to your all comments and those of other reviewers. We greatly appreciate the constructive feedback and all the comments, which have significantly improved the manuscript.

The table below addresses the issues that were raised and includes the page and line numbers corresponding to the track changes in the manuscript.

|  | **No.** | | **Section with comments** | **Reviewer’s comments** | **How the comments were addressed** | **Section, page/line where comments addressed** | |
| --- | --- | --- | --- | --- | --- | --- | --- |
|  | **Academic Editor** | | | | | | |
|  | |  | | - Please format the manuscript according to the journal requirements <https://journals.plos.org/plosone/s/submission-guidelines>. - Please refer to PLOS ONE downloadable sample files to ensure that your submission meets the journal formatting guidelines - Please ensure you have followed STROBE guidelines for cross-sectional studies - Pease make sure that ALL abbreviations are explained in the main text. Line 55 in the introduction - "ANC" abbreviation is not defined | Thank you.   - The manuscript was formatted according to the journal requirements. - Plos one sample file was used to meet the journal formatting guidelines. - The STROBE guidelines for cross-sectional studies were followed. - All abbreviations were cross-checked and explained in the main text. | Whole document  Whole document  Whole document  Page 4  Line 55 |  |
|  | **1.** | |  | Please ensure that your manuscript meets PLOS ONE's style requirements, including those for file naming. | 1. Thank you. The manuscript was formatted to meet PLOS ONE’s style requirements. | Whole document | |
|  | **2.** | |  | 2. Please amend your current ethics statement to address the following concerns:  a) Did participants provide their written or verbal informed consent to participate in this study? b) If consent was verbal, please explain i) why written consent was not obtained, ii) how you documented participant consent, and iii) whether the ethics committees/IRB approved this consent procedure | 2. Thank you for this comment.  The ethics statement was amended in the manuscript.  a) This study was a secondary analysis of aggregate data obtained through the RDHS 2019-20 survey. The study was registered and approved by the University of Rwanda-College of Medicine and Health Sciences Internal Review Board (CMHS/IRB/212/2021)  The original study, the RDHS 2019-20 population-based survey, obtained consent before the interview. Written informed consent was obtained from those who could read the consent form, and verbal consent from those who could not read.  b) i) and ii) Respondents from the original RDHS study who could not read gave verbal consent to participate, and this was documented in the original study.  iii) The survey protocol of the original study was reviewed and approved by the Rwanda National Ethics Committee (RNEC) and the ICF Institutional review board. | Page 7-8  Lines 140-146 | |
|  | **3.** | |  | In your Data Availability statement, you have not specified where the minimal data set underlying the results described in your manuscript can be found.  Upon re-submitting your revised manuscript, please upload your study’s minimal underlying data set as either Supporting Information files or to a stable, public repository and include the relevant URLs, DOIs, or accession numbers within your revised cover letter. | Thank you. The data availability statement has been amended through the manuscript specifying the link where the minimal data set may be found. Because this is a secondary data analysis of the RDHS, data are owned by a third-party organization it is not permissible to share with the public. However, the data is publicly available with permission from the MEASURE DHS Data Archive at ICF International and obtained upon request via the following link [www.measuredhsprogram.com](http://www.measuredhsprogram.com)  The third-party data underlying the results presented in the study are available from The DHS Program. Researchers can register on The DHS Program website (<https://dhsprogram.com/>) and request access to the DHS datasets. The 2019-2020 Rwanda Demographic Health Survey DHS data is available for download using the following link: (<https://dhsprogram.com/data/dataset/Rwanda_Standard-DHS_2019.cfm?flag=1>). The authors confirm they had no special access privileges. | Page 7  Lines  145-146  Page 18  Lines 303-305 | |

| **Reviewer #1: PONE-D-22-19638** | | | | |
| --- | --- | --- | --- | --- |
| **1.** | **Study design** | Regarding the study design, I understood (line no.99) that the current study used the data from RDHS (a cross sectional survey). If it is so, the current study cannot be a cross-sectional study, I feel. The study used part of data from 2019-20 RDHS, therefore, it could be a secondary analysis, as observed in a similar study conducted and published in 2016 by Rwabufigiri BN et al. (ref 18 of the current version of the manuscript) where they clearly mentioned about the study design. Please relook into their study design and rewrite accordingly. If my understanding is not correct, please clarify. | 1. Thank you for the comment.  The comments were addressed in the manuscript. | Page 6  Lines 99-101 |
| **2.** | **Study variables** | 2. Under study variables, the manuscript discussed that no. of visits was coded (line no. 121). But, the manuscript failed to interpret these findings. I am trying to think louder on this particular variable. Somehow, this variable may allow the policy makers to think about the bottlenecks in the delivery of adequate ANC services, thereby, they can plan some interventions like providing resources, IEC or awareness etc. For instance, even if X no of pregnant women completed at least four visits of ANC, whether all of these X subjects received adequate ANC (in terms of others components-time of first visit and receipt of the four components). I assume that authors included all these three components under one variable named ‘Adequate ANC’. However, results can be more appreciated if the manuscript provides these estimates as well. If you disagree of this point, please justify. If agree, kindly present all the missing data. | 2. Thank you for this feedback.  In the present study, receipt of Adequate ANC was coded as a dichotomous variable (yes/no). This variable was created by combining three different variables i) the number of ANC received, ii) the timing of the receipt of the first ANC, and 3) the total number of ANC services received. A woman is considered to have received adequate ANC if she has received at least four ANC, the first of which is in the first trimester and has received all four services of ANC (blood pressure and arm circumference measurements and blood and urine analysis). We have indicated this in the revised version of the manuscript. | Page 7  Lines 119-121 |
| **3.** | **Statistical analysis** | 3. Under the statistical analysis of methods section, kindly elaborate the variables (line no. 145) and factors that are considered in the bivariate analysis. Like demographics, socio economic characters etc. | 3. Thank you for the suggestion.  The variables were elaborated in the bivariate analysis, demographics and socio-economic characters were well detailed in the methods section. | Page 8  Lines  148-150 |
| **4.** | **Results section** | a.Kindly rewrite the title of Table 1. It would be better to mention these descriptive statistics in the methods sections rather than in the title. As these are the demographics and socio-economic characters of the study population, kindly relook into it. | a. Thank you for the suggestion.  The title of the tables were rewritten. | Page 9,10 |
|  |  | b. To bring the uniformity in the age groups and make them comparable, it is advisable to split the last group “35-49 years” into two groups as ‘35-44 years’ and ’45 years and above’. If agreed, the further calculations in the Table 2 and Table 3 will also be varied accordingly. | b. Thank you for the suggestion. The last group “35-49 years” was split into two groups for all tables (1, 2, 3) and related text. | Pages 9, 10, and 13 |
|  |  | c. When I go through the results, there is a slight confusion in the number of participants considered in the multivariate analysis. Throughout the manuscript, the study aimed at identifying the factor associated with the adequate ANC services. In the Table 1, it was already estimated that 27.6% of study population received adequate ANC. If author wants to represent the demographic and socio-economic characters of adequate ANC received population, the sample size cannot be 6039 (n) i.e., try to avoid the inadequate ANC population. If author wishes to represent characters of all (n=6039) participants, the title of Table 2 should not include the word ‘adequate ANC’. Indeed, my suggestion would be to merge the Table 1 and 2 and change the results interpretation accordingly. Kindly address. | c. We appreciate this feedback. The main purpose of Table 2 was to show the difference in the proportion of the receipt of adequate ANC by the participants’ characteristics. The confusion arises from using the word “Adequate ANC” in the title for Table 2. We have now replaced the title for Table 2 with “Characteristics of the study sample by ANC status” | Page 10 |
|  |  | d. The current study aimed to estimate the factors associated with adequate ANC, but not the ANC utilization in the study area, if I am not wrong. Therefore, total population (n=6039) cannot be used in multivariate analysis (provided in Table 3), when the Adequate ANC was found to be on 27.6% (n=1691). Accordingly, results, discussion and conclusion of the study may vary. I strongly suggest taking recommendation/expert opinion from Biostatistician on this matter. | d. Thanks for this feedback. Yes, we examined factors associated with the receipt of adequate ANC (coded as yes/no) as described above. As such, to run a logistic regression with this variable as our dependent variable, we need to have both groups of women, i.e., women who received adequate, ANC and the comparison group, i.e., women who did not receive adequate ANC. One of the authors of this study is an epidemiologist/biostatistician with an extensive experience in this type of analysis. To avoid confusion, we have changed the title for Table 3 to “Unadjusted and Multivariable-adjusted associations between characteristics of women and receipt of adequate ANC (N=6039).” | Page 13 |
|  |  | e. It is advisable to categorize birth order variable as 1, 2, 3 and 4 and above. Accordingly find the association. | e. Thank you for the suggestion. The birth order was categorized as advised for all tables (1, 2, 3) and related text. | Pages 9, 10 and 13 |
| **5.** | **Discussion section** | 5. In the discussion section, from 204-206, there is a problem in the references quoted. 18th reference is on post natal care. In line no. 206, I think, 17th reference to be quoted instead of 18th reference in the current version of manuscript. Kindly relook into these references (i.e.17, 18, &19) and address properly. Also kindly make necessary changes in the order of references as appropriate. | Thank you. The reference was removed and changes in the order were done appropriately. | Page 12  Line 206 |
|  | **Minor comments** | 1.Since the current study has not performed the correlation tests, the term co-variates in line no. 115, 129 may be replaced with variables | 1. Thank you. The term was replaced in the manuscript. | Page 6  Line 115,130 |
|  |  | 2. In Table 2, kindly relook into the percentages provided under the following variable- a. Age group & Education (calculation is not correct)  b. Health Insurance & Distance (please make it to 100%) | 2. Thank you. The calculation was relooked and corrected. | Page 10,Table 2 |
|  |  | 3. In line no. 204, references (supposed to be 17, 18) are in wrong order. | Thank you. The comments were addressed in the manuscript. | Page 12  Line 206 |
|  |  | 4. Suggestion to change the title: since antenatal care provides during pregnancy, slight corrections may be made in the title like Factors associated with adequate antenatal care among pregnant women in Rwanda......... If this study is not a primary study conducted by the authors, population-based cross-sectional study can be replaced with better terms to make it more appropriate. | Thank you for the suggestion. The title has been amended to make it more appropriate. | Page 1  Lines 2-4 |
|  |  | 5. References: strictly adhere to the journal guidelines. | Thank you. This was taken into account while revising the manuscript. | The whole document |

|  | **Reviewer #2** | |  |  |
| --- | --- | --- | --- | --- |
| **1** |  | Manuscript should be further revised in order to correct some typos and improve style | Thank you. The manuscript was revised, typos corrected, and style improved. | In submission system |
| **2** |  | Authors should add further details to discuss, at least briefly, the role of first-trimester screening for fetal aneuploidies, especially in advanced maternal age (authors may refer to: PMID: 27442264; PMID: 25027820). | Thank you for your suggestion. It is written in the further studies part. | Page 15-16  Lines 250-253 |
