# Supplementary material for: Factors associated with receipt of adequate antenatal care among women in Rwanda: A secondary analysis of the 2019–20 Rwanda Demographic and Health Survey
Source: PLoS One. 2023 Apr 20;18(4):e0284718. doi: 10.1371/journal.pone.0284718 (PMC10118085; doi:10.1371/journal.pone.0284718)
Supplement: Attachment — Submitted filename: Response to reviewers.docx [file pone.0284718.s002.docx]

#### Rebuttal letter

Dear Editor,

Thank you for taking the time to review our manuscript and allow us the opportunity to respond to your all comments and those of other reviewers. We greatly appreciate the constructive feedback and all the comments, which have significantly improved the manuscript.

The table below addresses the issues that were raised and includes the page and line numbers corresponding to the track changes in the manuscript.

| **No.** | **Section with comments** | **Reviewer’s comments** | **How the comments were addressed** | **Section, page/line where comments addressed** |
| --- | --- | --- | --- | --- |

| **Reviewer #1: PONE-D-22-19638** | | | | |
| --- | --- | --- | --- | --- |
| **1.** | **Results** | In the methodology, You mentioned about Chi square test under statistical analysis. However, no where it is mentioned in the results section. Have authors only considered for estimating p-value? Kindly justify. | 1. Thank you for the comment.  We now have this in the results section. Also, we have included the chi-square values and degrees of freedom in the text and table 2. | Page 10  Lines 169 -175 |
|  |  | Kindly re-looks into the interpretation of results at line 184-186. This result may be interpreted other way round. | 2. Thank you for the comment.  The results in line 184-186 are interpreted correctly. Compared to women who wanted the current pregnancy, the odds of having adequate ANC were significantly lower for women who wanted a child later (AOR 0.60: CI=0.52, 0.69; p=0.001) and those who never wanted a pregnancy (AOR 0.67: CI=0.55, 0.82; p<0.001). Women who perceived distance to a health facility as a big problem had lower odds of receiving adequate ANC than those who considered distance not a big problem (AOR 0.82: CI=0.70, 0.96; p=0.013). | Page 10  Lines 186-188 |
|  |  | In the Table-3 title, can we not use 'ANC utilization' instead of 'ANC Status'. Because the interpretation of results given in table-3 are all about receiving ANC. Kindly relook into it. | 3. Thank you for this feedback.  We have changed the title of table 3 to “Factors associated with receipt of adequate ANC.” This is consistent with the aim of the study. | Page 10 |
| **2.** | **References** | References were not written properly. Please strictly adhere to the journal author guidelines. For example, ref 11 is not written completely. There are other issues in rest of the reference. Kindly cross check all the reference again. | 2. Thank you for this feedback that helped to improve the reference list accordingly based on PLOS ONE guideline. | Page 18-20  Lines 304-437 |
