# Supplementary material for: Factors associated with receipt of adequate antenatal care among women in Rwanda: A secondary analysis of the 2019–20 Rwanda Demographic and Health Survey
Source: PLoS One. 2023 Apr 20;18(4):e0284718. doi: 10.1371/journal.pone.0284718 (PMC10118085; doi:10.1371/journal.pone.0284718)
Supplement: Attachment — Submitted filename: RESPON~1.DOC [file pone.0284718.s003.DOC]

#### Rebuttal letter

Dear Editor,

Thank you for taking the time to review our manuscript and allow us the opportunity to respond to your all comments and those of other reviewers. We greatly appreciate the constructive feedback and all the comments, which have significantly improved the manuscript.

The table below addresses the issues that were raised and includes the page and line numbers corresponding to the track changes in the manuscript.

| **No.** | **Section with comments** | **Reviewer’s comments** | **How the comments were addressed** | **Section, page/line where comments addressed** |
| --- | --- | --- | --- | --- |

| **Reviewer #1 PONE-D-22-19638R2** | | | | |
| --- | --- | --- | --- | --- |
| **1.** | **Methodology** | 1. In the Methodology, at line 138 under Ethics approval, a minor language correction is required. Kindly, you may use ‘which was’ instead of ‘and’ (before registered). | 1. Thank you for the comment. It has been addressed as suggested | Page 7  Line 138 |
| **2.** | **Results** | 2. In title of table-2, does the sub-heading ‘adequacy ANC received’ or ‘Adequacy of ANC received’? Please use appropriate sub-heading. | 2. Thank you for the comment.  We have used the appropriate sub-heading | Page 10 |
|  |  | 3. A suggestion from my side about a point that starts at Line 181 till 184 as follows – ‘Women from a middle and rich wealth indexes had higher odds of receiving adequate ANC ((AOR=1.24: CI=1.04-1.48; p=0.018) and (AOR=1.37: CI=1.16-1.61; p<0.001) respectively) compared to women from poor household wealth index’ | 3. Thank you for this feedback.  The suggestion was well received and re-written. | Page 11  Lines 181-184 |
|  |  | 4. Point starts at end of line no. 189 till 191 is misinterpreted, as per the results provided in Table-3 regarding distance to health facility. Kindly re-look into it. | Thank you for the comment. There was an error in labeling the reference group for distance to a health facility in table 3 reference group. This issue is now corrected. | Page 11  Line 189-192  Page 12 |
|  |  | 5. At line 261, current study is not directly providing recommendations on interventions. Perhaps, it helps in identifying the factors associated with ANC. Accordingly, it may help health care system to identify the interventions and implement them to improve ANC in the study area. If you agree, kindly re-write the statement (at end of line 261 and 262) | Thank you for the comment.  The statement was re-written. | Page 15  Lines 261-262 |
| **2.** | **References** | 6. References: Total references quoted in the text are 40, whereas under references sections, authors have listed 41. Please check and correct it | 2. Thank you for the comment.  References cross-checked and there were several systematic reviews we added the first author’s name to reduce confusion. | Page 18-20  Lines 304-437 |
|  |  | 7. Authors are also requested to adhere to Journal author guidelines while writing the reference. In most of the references, month was written. For example, look at ref. no. 4, 16, 17, 18 and others. Please cross check all the references again. | Thank you for this feedback that helped to improve the reference list based on PLOS ONE guidelines. | Page 20 |
